# Supplementary material for: The Role of Adhesion Molecules and Extracellular Vesicles in an In Vitro Model of the Blood–Brain Barrier for Metastatic Disease
Source: Cancers (Basel). 2023 Jun 3;15(11):3045. doi: 10.3390/cancers15113045 (PMC10252721; doi:10.3390/cancers15113045)
Supplement: Supplementary file 1 [file cancers-15-03045-s001.zip › cancers-2280160-supplementary.pdf]

Supplementary figures

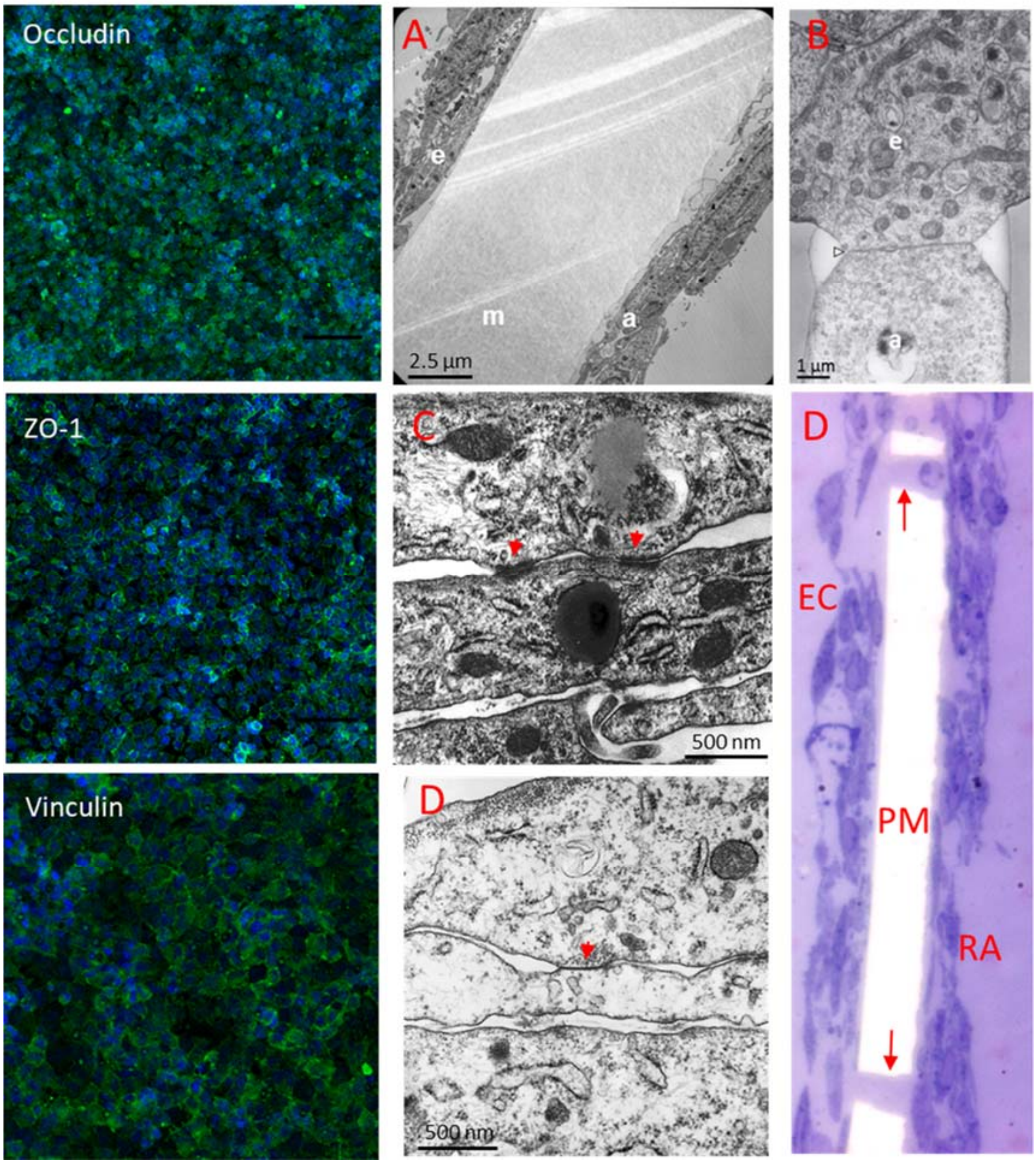

Supplementary figure S1: characterization of the BBB model using confocal microscopy (left panel) and electron microscopy (right panels, A-D) at day four from the cell culture start. On the left, the expression of occludin, zonula occludens-1 (ZO-1) focalized in membrane and vinculin with merge of DAPI nuclear staining (20xmagnification, 60 h of co-culture). On the right: panel A shows the polycarbonate membrane (m) coated from one side by HUVECs (e) and rat astrocytes on the other side (a). B: the image shows the contact between astrocytes (a) and endothelial cells (e) though an 8µm pore in the polycarbonate membrane (white arrow); C-D: tight junction between endothelial cells (red arrows);); E: each transmigration experiment was validated using a transwell insert specifically designed for the evaluation of HUVEC monolayer after 96 h of co-culture. Microscopic examination of 1 μm thick sections counterstained with toluidine blue verified the presence of astrocytes and the HUVEC monolayer (EC=endothelial cells; PM=polycarbonate membrane; RA= rat astrocytes; arrows indicate 8µm pores in the membrane).

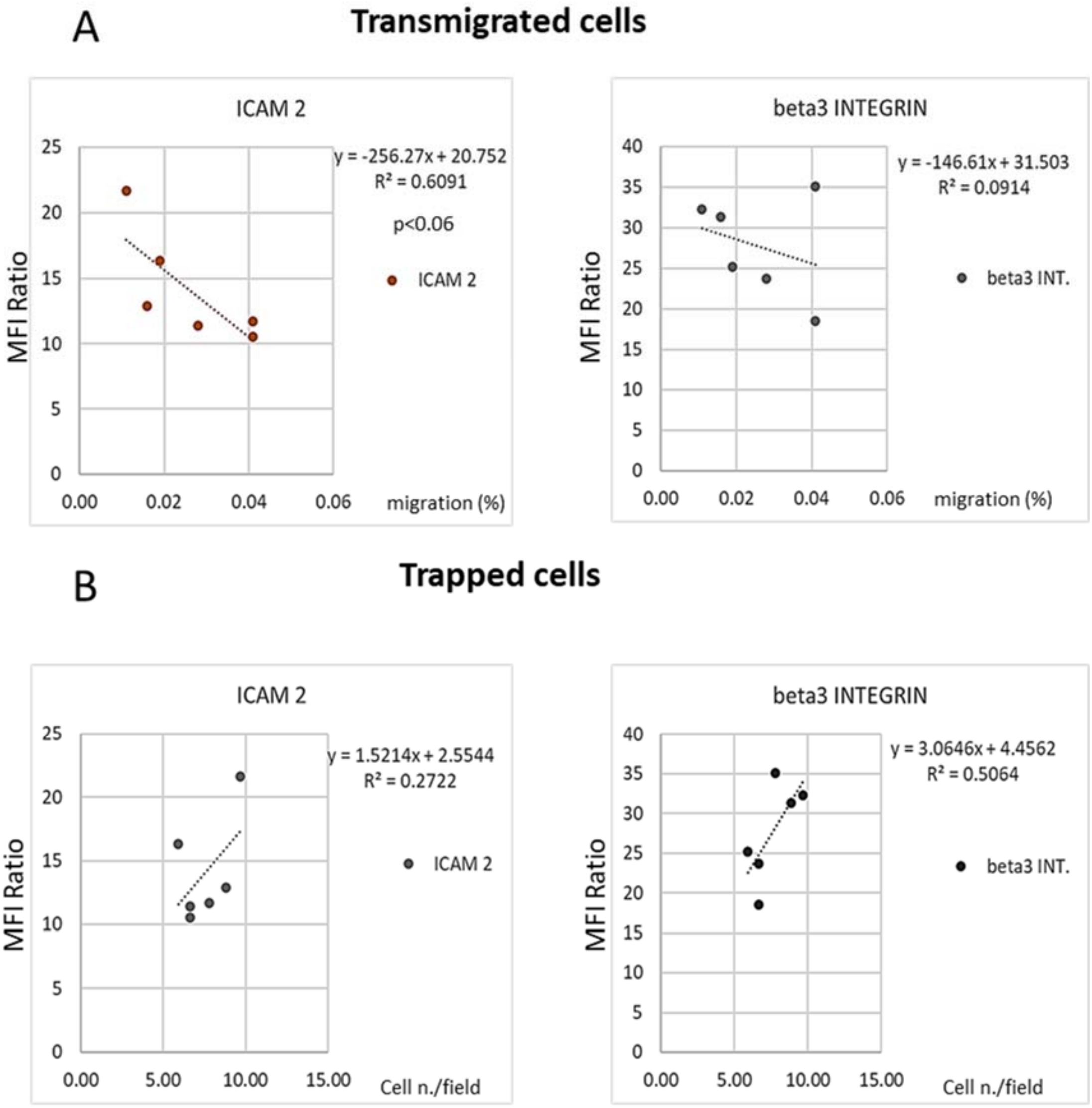

Supplementary figure S2: migration of each cell line, expressed as the percentage of cells counted in the bottom well of the transwell (panel A) or cells counted in the bottom side of the insert, was correlated to the MFI ratio. A trend to inverse correlation between adhesion molecules (ICAM2 and expression and trasmigrated cells was detected in all cell lines). On the other hand, the number of cells trapped in the insert were directly related with the level of adhesion molecules expression.

A

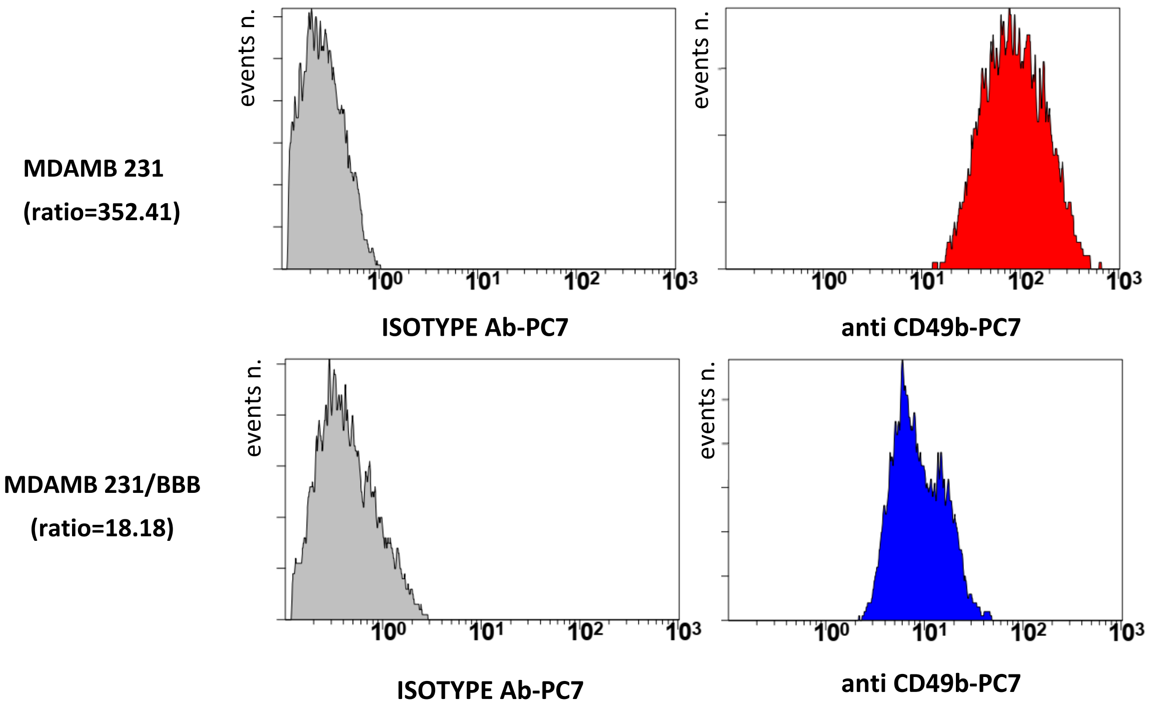

B

	A549	MCF7	MDA-MB231	H460
CCR7	ns	ns	ns	ns
ICAM 1	0.02	0.05	0.01	ns
α2 INT	0.05	0.05	0.02	ns
NCAM	ns	ns	ns	ns
MCAM	0.01	ns	0.02	ns
VCAM	ns	ns	ns	ns
β3 INT	ns	ns	ns	ns
ICAM 2	ns	ns	ns	ns

Supplementary figure S3: A) α2-integrin expression level in the original cell line MDAMB 231 and its subpopulation expanded from cells recovered in the bottom well of the transwell after the transmigration experiments (MDAMB 231-BBB) (see M&M). Mean fluorescence intensity (MFI) of the red histogram (MDAMB 231) is about 10 fold higher than that of the blue histogram (MDAMB 231/BBB) while the MFI of the isotype control is similar between the two cell lines (grey histograms). The ratio (in parentheses) between the MFI of specific antibody and MFI of isotype control is an index of the expression of the molecule. Data refers to a typical acquisition which include at least 10,000 events. B) p values (Student T test) obtained by the comparison between the expression of each adhesion molecules in the original cell line vs the cells expanded after transmigration.

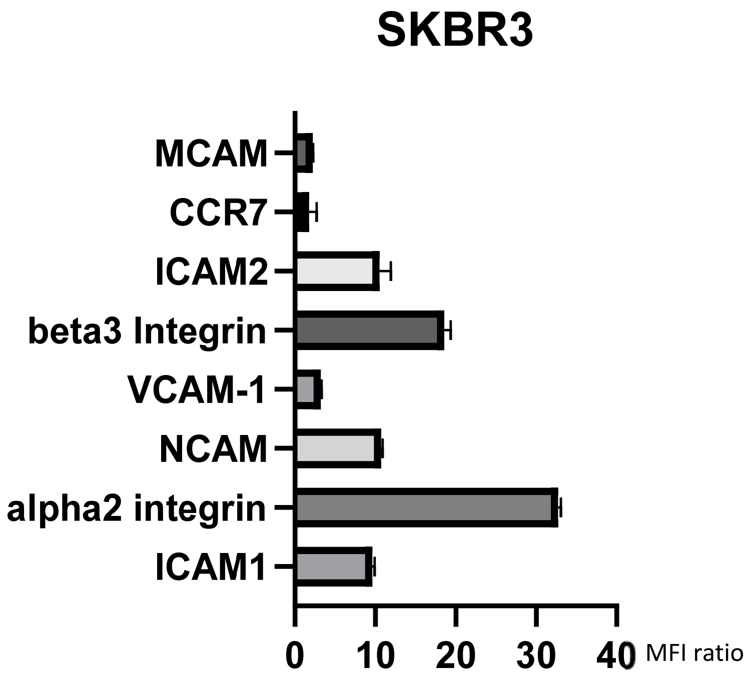

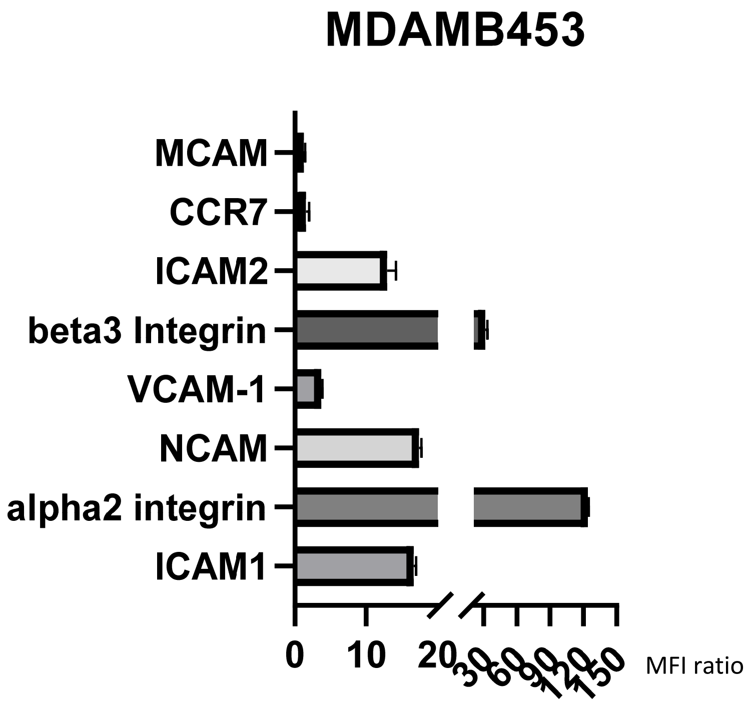

Supplementary figure S4: Adhesion molecules expression levels in SKBR3 and MDAMB453 cell lines as detected by flow cytometry. In these two cell lines we were not able to expand the population of cells recovered from the bottom side of the transwell. Data are expressed as MFI of specific antibody and isotype control. Date refers to at least three independent experiments.
